# Factors Associated With Mental Health Symptoms During the COVID-19 Pandemic in Hong Kong

**DOI:** 10.3389/fpsyt.2021.617397

**Published:** 2021-06-07

**Authors:** Sheng Zhi Zhao, Tzu Tsun Luk, Yongda Wu, Xue Weng, Janet Yuen Ha Wong, Man Ping Wang, Tai Hing Lam

**Affiliations:** ^1^School of Nursing, University of Hong Kong, Hong Kong, China; ^2^School of Public Health, University of Hong Kong, Hong Kong, China

**Keywords:** COVID-19, risk factors, mental health, unemployment, public health

## Abstract

**Background:** The COVID-19 pandemic has led to an increasing mental health burden. We examined the factors associated with mental health symptoms in Chinese general adults in Hong Kong.

**Methods:** We conducted a dual-frame (landline and mobile) survey on Chinese adults aged 18 years or older in April 2020. Shortage of preventive materials, perceptions of the outbreak (each item range 1–5), and reduction in income were assessed. Mental health symptoms measured included stress (Perceived Stress Scale-4, range 0–16), anxiety (General Anxiety Disorders-2, range 0–6, cutoff >2), and depressive symptoms (Patient Health Questionnaire-2, range 0–6, cutoff >2). Results were weighted by the general population distribution. Associations were analyzed by multivariable linear (for stress) and logistic (for anxiety and depressive symptoms) regression adjusting for sociodemographic and health-related covariates, including confirmed or in close contacts of confirmed cases, chronic disease, self-rated health, and smoking and alcohol drinking behavior.

**Results:** Of the 1,501 participants (52.5% female, 55.0 aged 30–59 years), the average stress score was 7.20 (SD 2.12). 218 (15.8%) and 206 (14.8%) participants had anxiety and depressive symptoms, respectively. Shortage in facemasks (20.8%), alcohol-based hand sanitizers (13.9%), and cleaning products (7.3%) was reported. Participants generally disagree with the perception of at risk of getting infected in the coming 6 months (mean 2.2, SD 1.1), but tended to agree with the perception of worry that the people around pose a threat to them (mean 3.6, SD 0.9) and the outbreak has greatly affected their daily life (mean 3.7, SD 0.9). 59.3% employed participants had income reduction and 6.2% had become unemployed since the outbreak. Stress, anxiety, and depressive symptoms were more prevalent in those with shortages of preventive materials and negative perceptions of the outbreak (all *P* < 0.05). Reduction in income and unemployment were associated with more mental health symptoms (all *P* < 0.05).

**Conclusions:** Shortage of preventive materials, negative perceptions, financial loss, and unemployment were prevalent during the outbreak and found in association with higher stress and more anxiety and depressive symptoms. Further research and urgent actions are warranted to relieve stress and promote mental health, targeting the many risk factors identified by our study.

## Introduction

The novel coronavirus disease 2019 (COVID-19) pandemic has led to an increase in mental health problems. Since the COVID-19 outbreak, a survey in China showed that 53.8% of the participants reported moderate to severe psychological distress (1). The prevalence of anxiety (5.3 vs. 31.6%) and depressive (6.0 vs. 27.9%) symptoms increased by about 4–5 times after the outbreak (2). In the USA, 13.6% of the adults experienced serious psychological distress in April 2020, compared with 3.9% in 2018 (3). Anxiety and depressive symptoms doubled in Hong Kong during the outbreak (4). Hong Kong had its first confirmed COVID-19 case on January 23, 2020. Public health interventions, including isolation, quarantine, contact tracing, work-from-home arrangements, school suspensions, shutdown of non-essential services, and social distancing, were shown to be effective measures to restrain the infection and mortality during the first 8 weeks such that only a few cases were reported per day (5). Changes in daily life and personal behaviors were substantial, such that avoiding crowded places and voluntarily wearing a facemask was reported by 85 and 98.8% of the participants, respectively, even without a complete lockdown (5). Following the influx of imported cases, local cluster outbreaks became more severe and Hong Kong had its second peak in late March with 1,035 confirmed cases by April 23, 2020.

The pandemic was spreading faster and wider than those of the past. Having confirmed or close contact with confirmed cases and living in the epicenter were found to be associated with mental health crisis during the pandemic (1, 2). Urgently adopted mitigation measures were accompanied by a disruption of daily life that was potentially associated with poor mental health both directly and indirectly (6–8). Exposure to the virus, quarantine, and isolation were directly linked to negative psychological health, especially for front-line medical workers and older adults (7, 9–11). Social distancing and personal protective measures were reported to be associated with stress, anxiety, and depressive symptoms (11). Other social consequences, including supplies of medical products, perceived affect, disrupted daily, study and work life, and financial insecurity, may have also impacted the mental health of the general population (6, 7, 12).

The use of facemasks, alcohol-based hand sanitizers, and other cleaning products (e.g., bleach) was advocated for breaking the chain of infection, which led to a surge in public demand for these preventive materials (1, 5, 13). Reports of shortages of medical protective materials caused enormous concern and led to panic buying and stockpiling during the local outbreak (14–16). Studies found that having sufficient medical resources was a protective factor against psychological distress (7, 16). Therefore, we hypothesized that lack of preventive material may contribute to higher stress and more mental health symptoms during the pandemic. Negative perceptions toward the pandemic may also exacerbate mental distress. Fear of contracting the virus was associated with poor mental well-being and lower quality of life and partially mediated the association between intolerance of uncertainty and depression (17, 18). The banning of social gathering, mandatory closure of schools, and suspension of non-essential productions and commercial activities disrupted most daily activities (6). Perceived at risk of getting infected and believe that the pandemic poses threats to one's health and significantly affected daily, study, and work life could possibly aggravate mental health symptoms, although the associations have remained understudied.

Border and transportation restrictions slowed global economic activity (19). Many transnational and local businesses, especially tourism and catering, were affected (19). International Labor Organization found an increase in the unemployment rate worldwide due to disruptions of socioeconomic activities (20). Delays in returning to work were found to increase risks of anxiety and depressive symptoms (2). Studies found that quarantine measures disproportionately affected those who had a lower household income (7, 11). Individuals with financial loss or unemployment since the pandemic may suffer from more mental health symptoms. Financial struggles, although indirectly associated with the pandemic and the mitigation measures, were suggested to be associated with mental health problems during the pandemic and, therefore, warrant further investigation (11).

Most current studies focus on well-known risk factors of mental distress (e.g., female gender, medical history, and poor self-rated health) and established stressors in a pandemic (e.g., being infected and quarantine) (7, 10). During this globally worsening pandemic, few studies have reported that the mental health of the general population was impacted by pandemic-related changes (2, 11, 21). By the end of March 2020, Hong Kong was recorded as one of the most heavily affected epicenters outside of Hubei, China. The mental health impact of the abrupt changes in population behavior observed in this developed urban city could be expected in other regions and countries even with sufficient medical resources. In this population-based cross-sectional study, we investigated potential risk factors, including perceived shortage of preventive materials, daily disruptions, and financial loss, and their association with stress and mental health (anxiety and depression) symptoms immediately after the peak of the second wave using a representative general adult sample in Hong Kong.

## Materials and Methods

### Study Design and Sampling

The Hong Kong COVID-19 Health Information Survey (CoVHInS) was conducted using a probability-based dual sampling frame of landline telephone and online mobile surveys on Chinese adults aged 18 and above. The survey was conducted from April 9 to April 23, 2020, ~2–4 weeks after the peak of the second wave of the COVID-19 outbreak in Hong Kong. Social Policy Research Limited, a reputable survey agency in Hong Kong, was commissioned to collect the data. The Institutional Review Board of the University of Hong Kong/Hospital Authority Hong Kong West Cluster (UW 20-238) approved of the study. Informed consent was obtained from all participants.

Details of the methods have been reported elsewhere (22). Briefly, a two-stage random sampling method was adopted for the landline survey. First, seed numbers were generated using the official's numbering plan for telecommunication services. A web-based Computer-Assisted Telephone Interview system (Web-CATI) was used to generate a random telephone numbers list for interviews. Second, of those we were able to contact, eligible household residents whose coming birthdays were closest to the interview day were selected (next birthday rule). All telephone interviewers completed a half-day training of COVID-19-related knowledge, contents of the questionnaire, sampling methods, and interviewing techniques. The questionnaire was pilot-tested on 10 participants to refine the items and procedure. Consistency checks were conducted during the interview to probe correct answers from the participants. To ensure the research fidelity, briefing and de-briefing sessions for interviewers were arranged, and rigorous quality and consistency checks were adopted (20% were checked). Each interview took ~25 min to complete. Among the 816 valid telephone numbers sampled, 500 participants completed the interview (response rate: 61.3%).

Participants of the online survey were sampled from a panel of residents' database. The survey panel was formed previously by responding to the invitation text messages sent to all the mobile phone numbers generated through the Numbering Plan for Telecommunication Services in Hong Kong. The numbers were provided by the Office of the Communications Authority, which covered over 90% of the Hong Kong residents. A total of 100,079 residents from diverse socio-economic background joined the panel. The survey agency adopted stratified random sampling by sex and age distribution and invited a random sample of panelist using text messages. Participants self-administered the questionnaire via the Web-CATI. Of the 1,623 panelists invited, 1,001 completed the survey (response rate of 61.7%).

### Measurements

We collected information related to sociodemographic characteristics (sex, age, and education attainment), COVID-19 diagnosis (at any point in time) or were in close contact with confirmed cases (no/yes), self-rated health (“excellent,” “very good,” “good,” “fair,” or “poor”) (23), diagnosed with chronic disease, such as hypertension, heart disease, or diabetes (none/any), and health-related behaviors which included tobacco smoking (never smoked, ex-smoker, or current smoker) and alcohol drinking (non-drinker, social drinker, monthly drinker, or daily drinker).

Shortage of preventive materials, including facemasks, alcohol-based hand sanitizers, and cleaning products (e.g., bleach), for the participants and their family members to use (at a household level) in the coming month was reported (no/yes). To assess perceptions of the COVID-19 pandemic, the participants were asked to what extent they agreed (“strongly disagree,” “disagree,” “neutral,” “agree,” and “strongly agree”) that (1) “I feel that I am at risk of getting infected in the coming 6 months;” (2) “I think people around me pose a threat to me;” (3) “I feel the outbreak greatly affected my daily life;” (4) “I feel difficult to study/work at home;” and (5) “I feel inefficient studying/working at home.” Homemakers and retirees were not asked about study/work at home. Reduction in income since the outbreak (no change, small reduction, reduction by half, larger reduction, or unemployed) were reported by participants who were economically active; while students, homemakers, and retirees were not asked the question.

Stress levels were assessed using the 4-item Perceived Stress Scale (PSS-4) (24). The PSS-4 measured the frequency of perceived ability to cope with existing stressors (two items) and lack of control and affective reactions (two items) in the preceding month (from early-March to mid-April 2020) on a Likert scale ranging from 0 (never) to 4 (very often). A higher total score indicates a higher perceived stress level (0–16). The Chinese version of PSS-4 has been validated in Hong Kong adults (25). The internal consistency of PSS-4 was satisfactory (Cronbach's alpha = 0.67) in our sample.

Anxiety and depressive symptoms were assessed using the 4-item Patient Health Questionnaire (PHQ-4) (26, 27). The scale had two core criteria for generalized anxiety disorder that screened for social panic and anxiety disorders (2-item General Anxiety Disorder, GAD-2), and two core diagnostic criteria for major depression disorders (2-item Patient Health Questionnaire, PHQ-2). Each item asked the frequency of anxiety/depressive symptoms in the past 2 weeks (from late-March to mid-April 2020) on a Likert scale ranging from 0 (not at all) to 3 (nearly every day). Subscales of the GAD-2 and PHQ-2 total scores ranged from 0 to 6 and a score ≥3 indicated the presence of anxiety and depressive symptoms, respectively. We previously validated the PHQ-2 in Hong Kong population (28). The internal consistency of GAD-2 (Cronbach's alpha = 0.81) and PHQ-2 (Cronbach's alpha = 0.81) were good in this study.

### Statistical Analysis

All data were weighted according to provisional figures obtained from the Census and Statistics Department on the sex, age, and education attainment distributions of Hong Kong general population in 2016. *T*-tests and χ^2^-tests were used to compare sociodemographic characteristics and other variables between the landline telephone and online self-administered samples. Multivariable regression was used to compare the stress, anxiety, and depressive symptoms by sociodemographic characteristics, COVID-19 cases, self-rated health, chronic disease, smoking, and drinking. The association of mental health symptoms with the shortage of preventive material, perceptions of the outbreak, and income change since the outbreak were separately analyzed by multivariable linear (for PSS-4) and logistic (for GAD-2 and PHQ-2) regression, adjusted for sex, age, and educational attainment (model 1). In model 2, we additionally included self-rated health, chronic disease, smoking, and alcohol drinking as covariates. We found that adopting personal protection [(1) wearing a surgical mask, (2) washing hands with alcohol-based sanitizers, (3) using alcohol to clean daily necessities, and (4) adding water to household drainage system, each reported with no/yes] and social distancing measures (e.g., avoiding crowded places, avoiding social gathering, keeping 1.5 m distance from others in public, and score ranging from 0 to 10 with higher scores indicating higher perceived compliance) were associated with lower risks of mental health symptoms in our previous report (11). In addition, we adjusted for these factors in model 3. All analysis was performed by using the STATA version/MP 15.1 (StataCorp., USA).

## Results

There were no significant differences in sociodemographic characteristics and health-related factors between participants from the landline (*n* = 500) and online (*n* = 1,001) samples (all *P* > 0.05) (Table 1). Of the 1,501 participants, 829 (52.5%) were women, 781 (55.0%) were aged between 30 and 59 years, and 1,254 (76.8%) had completed secondary or higher education. Forty (2.5%) participants were either diagnosed with COVID-19 or in close contact with confirmed cases. Four hundred and thirty-nine (28.4%) participants reported fair or poor health and 187 (15.0%) had a chronic disease. Prevalence of current smokers and daily drinkers were 18.0 and 2.8%, respectively. The average stress score was 7.20 (SD 2.12). Two hundred and eighteen (15.8%) and 206 (14.8%) participants had anxiety and depressive symptoms, respectively.

**Table 1:** Sociodemographic characteristics and health status by sampling method.

	**Sampling method**			**Combined (*****N*** **=** **1,501)**	
	**Landline telephone (*N* = 500)**	**Online self-administrated (*N* = 1,001)**	***P-*value**	**Unweighted**	**Weighted*^a^***
	***n* (%)**	***n* (%)**		***n* (%)**	**%**
**Sex**			0.08		
Male	208 (41.6)	464 (46.4)		672 (44.8)	47.5
Female	292 (58.4)	537 (53.6)		829 (55.2)	52.5
**Age, years**			0.11		
18–29	68 (13.6)	157 (15.7)		225 (15.0)	17.3
30–49	162 (32.4)	366 (36.6)		528 (35.2)	34.2
50–59	87 (17.4)	166 (16.6)		253 (16.9)	20.8
60–69	153 (30.6)	274 (27.4)		427 (28.4)	14.9
≥70	30 (6.0)	38 (3.8)		68 (4.5)	12.8
**Education attainment**			0.64		
≤ Primary	88 (17.6)	159 (15.9)		247 (16.5)	23.2
Secondary	287 (57.4)	577 (57.6)		864 (57.5)	45.4
Tertiary	125 (25.0)	265 (26.5)		390 (26.0)	31.4
**Confirmed cases or in close contact of confirmed cases**	11 (2.2)	29 (2.9)	0.43	40 (2.7)	2.5
**Self-rated health**			0.50		
Excellent	27 (5.4)	45 (4.5)		72 (4.8)	5.7
Very good	133 (26.6)	302 (30.2)		435 (29.0)	30.4
Good	187 (37.4)	368 (36.8)		555 (37.0)	35.5
Fair	143 (28.6)	273 (27.3)		416 (27.7)	26.5
Poor	10 (2.0)	13 (1.3)		23 (1.5)	1.9
**Any chronic diseases**	69 (13.8)	118 (11.8)	0.27	187 (12.5)	15.0
**Tobacco smoking**			0.41		
Never smoker	356 (71.2)	698 (69.7)		1054 (70.2)	71.5
Ex-smoker	48 (9.6)	119 (11.9)		167 (11.1)	10.5
Current smoker	96 (19.2)	184 (18.4)		280 (18.7)	18.0
**Alcohol drinking**			0.83		
Non-drinker	261 (52.2)	518 (51.8)		779 (51.9)	54.8
Social drinker	141 (28.2)	302 (30.2)		443 (29.5)	28.0
Monthly drinker	83 (16.6)	152 (15.2)		235 (15.7)	14.4
Daily drinker	15 (3.0)	29 (2.9)		44 (2.9)	2.8
**Perceived stress, mean (SD)*^b^***	7.1 (2.1)	7.3 (2.1)	0.98	7.2 (2.1)	7.2 (2.1)
**Anxiety symptoms*^c^***	65 (13.0)	153 (15.3)	0.24	218 (14.5)	15.8
**Depressive symptoms*^d^***	60 (12.0)	146 (14.6)	0.17	206 (13.7)	14.8

a *Weighted by sex, age, educational attainment distributions of Hong Kong general population in 2016*.

b *4-item Perceived Stress Scale, range 0–16, higher score indicating higher stress level*.

c *2-item General Anxiety Disorder, range 0–6, a score of ≥3 indicates positive for anxiety symptoms*.

d *2-item Patient Health Questionnaire; range 0–6, a **score** of ≥3 indicates positive for depressive symptoms*.

Table 2 presents the shortage in preventive materials including facemasks (20.8%), alcohol-based hand sanitizers (13.9%), and cleaning products (7.3%). Of the 1,501 participants, 349 (24.1%) reported shortage in at least one type of preventive material. Participants, generally, reported that they did not believe that they were at risk of getting infected in the following 6 months (mean 2.2, SD 1.1), but agreed with the perception of worry that the people around posed a threat to them (mean 3.6, SD 0.9) and the outbreak had greatly affected their daily life (mean 3.7, SD 0.9). The mean score of perceived difficult to and less efficient studying or working at home was 3.1 (SD 1.1) and 3.2 (SD 1.1), respectively. Of the participants, 59.3% (626/1,043) experienced different levels of income reduction and 6.1% had become unemployed since the outbreak.

**Table 2:** Shortage of preventive materials, perception of the outbreak, and change in income, smoking, and alcohol consumption since the outbreak.

	**Crude *n* (%)**	**Weighted^a^ %**
**Shortage of preventive materials**		
Facemasks	294 (19.6)	20.8
Alcohol-based hand sanitizers	194 (12.9)	13.9
Cleaning products (e.g., bleach)	90 (6.0)	7.3
**No. of types of shortage materials**		
None	1,152 (76.8)	75.9
1	177 (11.8)	11.6
2	115 (7.7)	7.1
3	57 (3.8)	5.4
**Perceptions of the outbreak, mean (SD)^b^**		
I feel that I am at risk of getting infected in the coming 6 months	2.1 (1.1)	2.2 (1.1)
I think people around me pose a threat to me	3.6 (0.9)	3.6 (0.9)
I feel the outbreak greatly affected my daily life	3.7 (0.9)	3.7 (0.9)
I feel difficult to study/work at home	3.2 (1.1)	3.1 (1.1)
I feel inefficient studying/working at home	3.2 (1.1)	3.2 (1.1)
**Change in income since the outbreak (*****n*** **=** **1,043)^c^**		
No change	347 (33.3)	34.5
Small reduction	323 (31.0)	29.9
Reduction by half	141 (13.5)	13.1
Large reduction	162 (15.5)	16.3
Unemployed	70 (6.7)	6.2

a *Weighted by sex, age, educational attainment distributions of Hong Kong general population in 2016*.

b *Range: 1 “strongly disagree” to 5 “strongly agree.”*

c *Excluded students, homemakers, and retirees*.

Table 3 shows that older age and higher education attainment were associated with lower stress score (all *P* for trend, which tested for the dose–response effect <0.001). Confirmed cases or close contacts with confirmed cases were associated with more anxiety (adjusted odds ratio 1.47, 95% CI 0.66, 3.28) and depressive (adjusted odds ratio 1.86, 95% CI 0.86, 4.04) symptoms. Poor self-rated health was associated with a greater stress score (adjusted β 3.61, 95% CI 2.66, 4.57) and more anxiety (adjusted odds ratio 10.71, 95% CI 3.48, 33.00) and depressive symptoms (adjusted odds ratio 11.76, 95% CI 3.78, 36.59). Participants with chronic diseases had a greater stress score (adjusted β 0.50, 95% CI 0.17, 0.85). Currently smoking and drinking daily were also associated with a greater stress score and more anxiety and depression symptoms (all *P* < 0.01).

**Table 3:** Mental health symptoms by sociodemographic factors, self-rated health, chronic disease, tobacco smoking, and alcohol drinking.

	**Stress score**	**Anxiety symptoms**	**Depressive symptoms**
	**Adjusted β^a^ (95% CI)**	**Adjusted OR^a^ (95% CI)**	**Adjusted OR^a^ (95% CI)**
**Sex**			
Male	–	–	–
Female	0.03 (−0.19, 0.25)	1.18 (0.86, 1.63)	1.18 (0.85, 1.64)
**Age, years**			
18–29	–	–	–
30–49	−0.65 (−0.96, −0.34)^***^	0.82 (0.53, 1.26)	0.82 (0.53, 1.27)
50–59	−0.96 (−1.33, −0.59)^***^	0.84 (0.50, 1.41)	0.61 (0.35, 1.05)
60–69	−1.14 (−1.52, −0.75)^***^	0.56 (0.32, 0.99)^*^	0.41 (0.23, 0.74)^**^
≥70	−1.98 (−2.59, −1.37)^***^	0.85 (0.37, 1.98)	0.65 (0.27, 1.56)
*P for trend*	<0.001	0.13	0.006
**Education attainment**			
≤ Primary	–	–	–
Secondary	−0.68 (−0.99, −0.37)^***^	1.14 (0.71, 1.84)	0.93 (0.57, 1.53)
Tertiary	−0.91 (−1.30, −0.51)^***^	1.39 (0.77, 2.51)	1.05 (0.57, 1.93)
*P for trend*	<0.001	0.23	0.73
**Confirmed cases or in close contact of confirmed cases**	0.26 (−0.36, 0.88)	1.47 (0.66, 3.28)	1.86 (0.86, 4.04)
**Self-rated health**			
Excellent	–	–	–
Very good	0.76 (0.27, 1.26)^**^	1.08 (0.52, 2.22)	0.98 (0.47, 2.02)
Good	1.39 (0.91, 1.89)^***^	0.84 (0.41, 1.74)	0.86 (0.41, 1.78)
Fair	2.00 (1.49, 2.51)^***^	1.40 (0.67, 2.95)	1.23 (0.58, 2.60)
Poor	3.61 (2.66, 4.57)^***^	10.71 (3.48, 33.00)^***^	11.76 (3.78, 36.59)^***^
*P for trend*	<0.001	0.02	0.02
**Any chronic disease**	0.51 (0.17, 0.85)^**^	1.27 (0.79, 2.02)	1.33 (0.82, 2.17)
**Tobacco smoking**			
Non-smoker	–	–	–
Ex-smoker	−0.38 (−0.72, −0.03)^*^	0.71 (0.39, 1.27)	0.72 (0.38, 1.36)
Current smoker	0.54 (0.26, 0.82)^***^	1.84 (1.27, 2.67)^***^	2.04 (1.40, 2.96)^***^
**Alcohol drinking**			
Non-drinker	–	–	–
Social drinker	−0.28 (−0.51, −0.05)^*^	0.86 (0.60, 1.21)	0.97 (0.69, 1.38)
Monthly drinker	0.03 (−0.27, 0.33)	1.00 (0.65, 1.55)	0.87 (0.55, 1.39)
Daily drinker	0.99 (0.38, 1.60)^***^	3.60 (1.85, 7.02)^***^	2.81 (1.40, 5.66)^**^

*OR, odds ratio. P for trend: test of the dose–response effect in the association*.

a *Each mutually adjusted for other variables above*.

* *P < 0.05*,

** *P < 0.01*,

*** *P < 0.001*.

Table 4 shows that stress score and anxiety and depressive symptoms were higher among those who had a shortage in preventive materials, adjusted for sociodemographic factors (all *P* < 0.001). Dose–response associations were found among the number of types of materials in shortage and stress, anxiety, and depression symptoms (all *P* for trend < 0.001). Negative perceptions of the outbreak (all *P* < 0.001) and reduction of income since the outbreak (all *P* for trend < 0.001) were all significantly associated with stress, anxiety, and depression symptoms. The stress levels (adjusted β 1.69, 95% CI 0.14, 2.24), and risks for anxiety (adjusted odds ratio 5.94, 95% CI 2.93, 12.04) and depressive (adjusted odds ratio 3.94, 95% CI 2.03, 7.66) symptoms were largely increased among those who had become unemployed during the pandemic. These associations were similar after we additionally adjusted for self-rated health, chronic disease, smoking and alcohol consumption behaviors, or personal protection and social distancing measures (Supplementary Table).

**Table 4:** Mental health symptoms by shortage of preventive materials, perceptions of the outbreak, and change in income, smoking, and alcohol consumption since the outbreak.

	**Stress score adjusted** **β** **(95% CI)**		**Anxiety symptom adjusted** **(OR 95% CI)**		**Depressive symptom adjusted** **(OR 95% CI)**	
	**Model 1^a^**	**Model 2^b^**	**Model 1^a^**	**Model 2^b^**	**Model 1^a^**	**Model 2^b^**
**Shortage of preventive materials**						
Facemasks	0.95 (0.69, 1.21)^***^	0.79 (0.53, 1.04)^***^	2.67 (1.93, 3.68)^***^	2.52 (1.82, 3.50)^***^	2.64 (1.90, 3.68)^***^	2.51 (1.79, 3.52)^***^
Alcohol-based hand sanitizers	1.30 (0.99, 1.61)^***^	1.13 (0.83, 1.43)^***^	2.30 (1.58, 3.34)^***^	1.65 (1.02, 2.66)^*^	2.41 (1.64, 3.53)^***^	2.22 (1.50, 3.27)^**^
Cleaning products (e.g., bleach)	0.83 (0.39, 1.27)^***^	0.72 (0.30, 1.14)^***^	3.81 (2.39, 6.06)^***^	2.53 (1.39, 4.61)^**^	3.39 (2.11, 5.46)^***^	3.26 (2.01, 5.29)^***^
**No. of types of shortage materials**						
None	–	–	–	–	–	
1	0.60 (0.28, 0.92)^***^	0.41 (0.03, 0.78)^*^	2.07 (1.38, 2.11)^***^	1.74 (1.07, 2.83)^*^	1.82 (1.19, 2.79)^**^	1.78 (1.16, 2.74)^**^
2	1.39 (1.00, 1.78)^***^	1.01 (0.52, 1.49)^***^	1.59 (0.94, 2.71)	1.23 (0.65, 2.33)	2.00 (1.19, 3.35)^**^	1.86 (1.10, 3.13)^*^
3	1.29 (0.74, 1.83)^***^	1.08 (0.39, 1.77)^**^	7.17 (4.07, 12.64)^***^	4.32 (2.03, 9.17)^***^	6.05 (3.40,10.77)^***^	5.62 (3.11, 10.13)^***^
*P for trend*	<0.001	<0.001	<0.001	<0.001	<0.001	<0.001
**Perceptions of the outbreak**						
I feel at risk of getting infected in the coming 6 months	0.18 (0.08, 0.28)^***^	0.12 (0.03, 0.22)^*^	1.56 (1.37, 1.78)^***^	1.54 (1.35, 1.76)^***^	1.42 (1.24, 1.62)^***^	1.39 (1.21, 1.59)^***^
I think people around me pose a threat to me	0.32 (0.20, 0.44)^***^	0.29 (0.17, 0.40)^***^	1.47 (1.24, 1.74)^***^	1.46 (1.23, 1.73)^***^	1.63 (1.37, 1.95)^***^	1.62 (1.35, 1.93)^***^
I feel the outbreak greatly affected daily life	0.31 (0.19, 0.42)^***^	0.27 (0.15, 0.38)^***^	1.38 (1.17, 1.64)^***^	1.35 (1.14, 1.60)^***^	1.41 (1.19, 1.68)^***^	1.38 (1.16, 1.65)^***^
I feel difficult to study/work at home	0.27 (0.16, 0.38)^***^	0.25 (0.14, 0.35)^***^	1.60 (1.37, 1.87)^***^	1.59 (1.36, 1.87)^***^	1.41 (1.21, 1.64)^***^	1.39 (1.19, 1.63)^***^
I feel inefficient studying/working at home	0.33 (0.22, 0.45)^***^	0.28 (0.17, 0.39)^***^	1.62 (1.38, 1.91)^***^	1.59 (1.35, 1.88)^***^	1.71 (1.44, 2.02)^***^	1.68 (1.41, 1.99)^***^
**Change in income since the outbreak^c^**						
No change	–	–	–	–	–	–
Small reduction	0.44 (0.11, 0.76)^**^	0.49 (0.18, 0.80)^**^	3.41 (2.00, 5.82)^***^	3.55 (2.07, 6.08)^***^	1.84 (0.23, 3.01)^*^	1.87 (1.13, 3.07)^*^
Reduction by half	0.55 (0.14, 0.97)^**^	0.61 (0.22, 1.01)^**^	4.30 (2.35, 7.90)^***^	4.52 (2.45, 8.35)^***^	2.62 (1.49, 4.63)^***^	2.68 (1.51, 4.76)^***^
Large reduction	0.81 (0.41, 1.21)^***^	0.80 (0.42, 1.19)^***^	4.52 (2.50, 8.16)^***^	4.43 (2.43, 8.07)^***^	2.47 (1.41, 4.32)^***^	2.32 (1.32, 4.10)^**^
Unemployed	1.69 (0.14, 2.24)^***^	1.61 (1.08, 2.14)^***^	5.94 (2.93, 12.04)^***^	5.61 (2.74, 11.51)^***^	3.94 (2.03, 7.66)^***^	3.53 (1.79, 6.96)^***^
*P for trend*	<0.001	<0.001	<0.001	<0.001	<0.001	<0.001

*OR, odds ratio. P for trend: test of the dose–response effect in the association*.

a *Regression model 1: adjusted for sociodemographic variables including sex, age, and education attainment*.

b *Regression model 2: additionally adjusted for self-rated health, chronic disease, smoking, and alcohol drinking*.

c *Excluded students, homemakers, and retirees*.

* *P < 0.05*,

** *P < 0.01*,

*** *P < 0.001*.

## Discussion

Our results provide the first evidence that shortage of preventive materials (facemasks, alcohol-based hand sanitizers, and other cleaning products) and negative perceptions of the outbreak are associated with stress and mental health (anxiety and depressive) symptoms. People experiencing financial loss and unemployment have more mental health symptoms after adjusting for sociodemographic and health-related factors. Our findings are consistent with previous studies that reported mental distress levels were influenced by the availability of medical resources, locally (10). Shortage of preventive materials was associated with higher risks for mental health symptoms. Since the initial outbreak in Wuhan, China, the fear and worry for infection led to early and voluntary mass masking in Hong Kong (5). The perceived risks of supply disruption and mass hoarding could exacerbate the stress and anxiety from personal insecurity and an uncertain future (29, 30). The situation could be worse in other countries or regions with unstable material supplies and serious outbreaks. With lower internet access and literacy and inflated prices, the elderly and poor experience more frustration in purchasing material (31, 32). Lack of facemasks was found in 9.8% of the participants aged 18–29 but increased to 38.4% in those aged 70 years and above. More participants who had become unemployed reported lack of facemasks than those who were employed (40.0 vs. 9.8%). Accessibility to preventive materials and health service resources for the most vulnerable should be further strengthened and improved. Regulating the supply chain, suppressing misinformation that exacerbates panic buying and hoarding, and ensuring the supply of protective equipment to the most vulnerable groups may promote community prevention and improve mental health during the pandemic (32).

As expected, those who had negative perceptions of the outbreak, including perceived risks of getting infected, worry that people around may pose a threat to oneself, and perceived great impact on daily life, were more likely to experience mental distress during the pandemic. With schools suspended and many companies and organizations changing to work from home, perceived difficulty and inefficiency of studying or working at home would add extra stress. A previous study in mainland China showed that those who worked at home had a higher risk of anxiety and depressive symptoms when compared with those who had returned to their workplace (2). Since the pandemic, two-thirds of our participants had incurred financial loss. Consistent with the literature (33, 34), we found that financial loss and unemployment since the outbreak were strongly and independently associated with increased mental health symptoms. Epicenters with growing outbreaks and continuous infection control policies may witness a delayed return of daily routine and experience larger impact.

More mental health symptoms in participants with poor self-rated health and a comorbid chronic disease was likely due to fear of COVID-19 and interruption or delay in treatment. Similarly, smokers and drinkers had more mental health symptoms. Smoking was related to the expression of ACE2 (receptor for SARS-CoV-2) for a higher COVID-19 severity (35), and alcohol drinking was linked to the COVID-19 cluster outbreaks in bars and a potential risk factor for disease severity and ICU admission (36). Older adults, however, reported less stress possibly attributable to less work-related essential tasks and daily life disruption.

The associations we identified support the need for specific psychological assistance for those with negative perceptions, financial loss, and unemployment during the pandemic. Accelerating the local infection control and facilitating the restructuring of daily, study, and work-life is needed to enhance mental well-being. Social and financial support for the most underprivileged people is urgently needed (37).

## Limitation

This study had several limitations. First, causal relations cannot be confirmed in cross-sectional studies. Second, unmeasured, or residual confounding could not be excluded. Third, use of self-reported measures is subject to recall errors. Clinical psychological screening may require rigorous measures but are currently hard to achieve. Fourth, non-response bias could not be excluded. To improve represntativeness, we weighted the data by sex, age, and education of the general population. The estimates computed by using weighted and unweighted data were also very similar. Finally, our study only provided a snapshot of immediate mental health responses following the COVID-19 outbreak in Hong Kong, which may evolve with the development of the pandemic, balance of the supply system, and the public health interventions. Longitudinal mental health impact on the general population and vulnerable groups needs further investigation.

## Conclusion

Shortage of preventive materials, negative perceptions, financial loss, and unemployment were prevalent during the outbreak and found to be associated with higher stress and more anxiety and depressive symptoms. Further research and urgent actions are warranted to relieve stress and promote mental health, targeting the many risk factors identified by our study.

## Data Availability Statement

The original contributions presented in the study are included in the article/Supplementary Material, further inquiries can be directed to the corresponding author.

## Ethics Statement

The studies involving human participants were reviewed and approved by The Institutional Review Board (IRB) of the University of Hong Kong/Hospital Authority Hong Kong West Cluster. The patients/participants provided their written informed consent to participate in this study.

## Author Contributions

SZ, JW, and MW conceived and designed the study. SZ and MW did the statistical analysis, wrote the first draft of the article, accountable for the accuracy, and integrity of the study. JW and MW supervised the study. All authors interpreted the data, participated in the critical review of the report, and provided final approval for publication submission.

## Conflict of Interest

The authors declare that the research was conducted in the absence of any commercial or financial relationships that could be construed as a potential conflict of interest.

## References

[1] Wang C Pan R Wan X Tan Y Xu L Ho CS. Immediate psychological responses and associated factors during the initial stage of the 2019 coronavirus disease (COVID-19) epidemic among the general population in China. Int J Environ Res Public Health. (2020) 17:1729. 10.3390/ijerph17051729 32155789 PMC7084952

[2] Shi L Lu Z-A Que J-Y Huang X-L Liu L Ran M-S. Prevalence of and risk factors associated with mental health symptoms among the general population in China during the coronavirus disease 2019 pandemic. JAMA Netw Open. (2020) 3:e2014053. 10.1001/jamanetworkopen.2020.14053 32609353 PMC7330717

[3] McGinty EE Presskreischer R Han H Barry CL. Psychological distress and loneliness reported by US adults in 2018 and April 2020. JAMA. (2020) 324:93–4. 10.1001/jama.2020.9740 32492088 PMC7270868

[4] Choi EPH Hui BPH Wan EYF. Depression and anxiety in Hong Kong during COVID-19. Int J Environ Res Public Health. (2020) 17:3740. 10.3390/ijerph17103740 32466251 PMC7277420

[5] Cowling BJ Ali ST Ng TWY Tsang TKL Li JCM Fong MW. Impact assessment of non-pharmaceutical interventions against coronavirus disease 2019 and influenza in Hong Kong: an observational study. Lancet Public Health. (2020) 5:e279–88. 10.1016/S2468-2667(20)30090-6 32311320 PMC7164922

[6] Pfefferbaum B North CS. Mental health and the covid-19 pandemic. N Engl J Med. (2020) 383:510–2. 10.1056/NEJMp2008017 32283003

[7] Vindegaard N Benros ME. COVID-19 pandemic and mental health consequences: systematic review of the current evidence. Brain Behav Immun. (2020) 89:531–42. 10.1016/j.bbi.2020.05.048 32485289 PMC7260522

[8] Dong L Bouey J. Public Mental Health Crisis during COVID-19 Pandemic, China. Emerg Infect Dis J. (2020) 26:1616–8. 10.3201/eid2607.202407 32202993 PMC7323564

[9] Brooks SK Webster RK Smith LE Woodland L Wessely S Greenberg N. The psychological impact of quarantine and how to reduce it: rapid review of the evidence. Lancet. (2020) 395:912–20. 10.1016/S0140-6736(20)30460-8 32112714 PMC7158942

[10] Luo M Guo L Yu M Jiang W Wang H. The psychological and mental impact of coronavirus disease 2019 (COVID-19) on medical staff and general public—a systematic review and meta-analysis. Psychiatry Res. (2020) 291:113190. 10.1016/j.psychres.2020.113190 32563745 PMC7276119

[11] Wang Y Shi L Que J Lu Q Lu Z Xu Y. The impact of quarantine on mental health status among general population in China during the COVID-19 pandemic. Mol Psychiatry. (2021). 10.1038/s41380-021-01019-y. [Epub ahead of print]. 33483692 PMC7821451

[12] Holmes EA O'Connor RC Perry VH Tracey I Wessely S Arseneault L. Multidisciplinary research priorities for the COVID-19 pandemic: a call for action for mental health science. Lancet Psychiatry. (2020) 76:547–60. 10.1016/S2215-0366(20)30168-1 32304649 PMC7159850

[13] Cheng KK Lam TH Leung CC. Wearing face masks in the community during the COVID-19 pandemic: altruism and solidarity. Lancet. (2020). 10.1016/S0140-6736(20)30918-1. [Epub ahead of print]. 32305074 PMC7162638

[14] Missoni E Armocida B Formenti B. Face masks for all and all for face masks in the COVID-19 pandemic: community level production to face the global shortage and shorten the epidemic. Disaster Med Public Health Prep. (2021) 15:e29–33. 10.1017/dmp.2020.207 32576310 PMC7371839

[15] Leung CC Lam TH Cheng KK. Mass masking in the COVID-19 epidemic: people need guidance. Lancet. (2020) 395:945. 10.1016/S0140-6736(20)30520-1 32142626 PMC7133583

[16] Tai Y-L Chi H Chiu N-C Tseng C-Y Huang Y-N Lin C-Y. The effect of a name-based mask rationing plan in Taiwan on public anxiety regarding a mask shortage during the COVID-19 pandemic: observational study. JMIR Form Res. (2021) 5:e21409. 10.2196/21409 33400678 PMC7837388

[17] Alyami M de Albuquerque JV Krägeloh CU Alyami H Henning MA. Effects of fear of COVID-19 on mental well-being and quality of life among saudi adults: a path analysis. Saudi J Med Med Sci. (2021) 9:24–30. 10.4103/sjmms.sjmms_630_20 33519340 PMC7839565

[18] Voitsidis P Nikopoulou VA Holeva V Parlapani E Sereslis K Tsipropoulou V. The mediating role of fear of COVID-19 in the relationship between intolerance of uncertainty and depression. Psychol Psychother. (2020). 10.1111/papt.12315. [Epub ahead of print]. 33216444 PMC7753422

[19] Baldwin R di Mauro BW. Economics in the Time of COVID-19. London: CEPR Press (2020).

[20] Almost 25 Million Jobs Could be Lost Worldwide as a Result of COVID-19 Says ILO. (2020). Available online at: http://www.ilo.org/global/about-the-ilo/newsroom/news/WCMS_738742/langen/index.htm (accessed August 13, 2020).

[21] Ran M-S Gao R Lin J-X Zhang TM Chan SKW Deng XP. The impacts of COVID-19 outbreak on mental health in general population in different areas in China. Psychol Med. (2020). 10.1017/S0033291720004717. [Epub ahead of print]. 33298220

[22] Zhao SZ Wong JYH Wu Y Choi EPH Wang MP Lam TH. Social distancing compliance under COVID-19 pandemic and mental health impacts: a population-based study. Int J Environ Res Public Health. (2020) 17:6692. 10.3390/ijerph17186692 32937929 PMC7560229

[23] Idler EL Benyamini Y. Self-rated health and mortality: a review of twenty-seven community studies. J Health Soc Behav. (1997) 38:21–37. 10.2307/2955359 9097506

[24] Cohen S Kamarck T Mermelstein R. A global measure of perceived stress. J Health Soc Behav. (1983) 24:385–96. 10.2307/2136404 6668417

[25] Leung DY Lam T-H Chan SS. Three versions of perceived stress scale: validation in a sample of Chinese cardiac patients who smoke. BMC Public Health. (2010) 10:513. 10.1186/1471-2458-10-513 20735860 PMC2939644

[26] Kroenke K Spitzer RL Williams JBW Löwe B. An ultra-brief screening scale for anxiety and depression: the PHQ-4. Psychosomatics. (2009) 50:613–21. 10.1016/S0033-3182(09)70864-3 19996233

[27] Löwe B Wahl I Rose M Spitzer C Glaesmer H Wingenfeld K. A 4-item measure of depression and anxiety: validation and standardization of the Patient Health Questionnaire-4 (PHQ-4) in the general population. J Affect Disord. (2010) 122:86–95. 10.1016/j.jad.2009.06.019 19616305

[28] Yu X Stewart SM Wong PTK Lam TH. Screening for depression with the patient health questionnaire-2 (PHQ-2) among the general population in Hong Kong. J Affect Disord. (2011) 134:444–7. 10.1016/j.jad.2011.05.007 21665288

[29] Zheng R Shou B Yang J. Supply disruption management under consumer panic buying and social learning effects. Omega. (2021) 101:102238. 10.1016/j.omega.2020.102238

[30] Arafat SMY Kar SK Menon V Kaliamoorthy C Mukherjee S Alradie-Mohamed A. Panic buying: an insight from the content analysis of media reports during COVID-19 pandemic. Neurol Psychiatry Brain Res. (2020) 37:100–3. 10.1016/j.npbr.2020.07.002 32834528 PMC7365627

[31] The Lancet. COVID-19: fighting panic with information. Lancet. (2020) 395:537. 10.1016/S0140-6736(20)30379-2 32087777 PMC7138040

[32] Ranney ML Griffeth V Jha AK. Critical supply shortages—the need for ventilators and personal protective equipment during the covid-19 pandemic. N Engl J Med. (2020) 382:e41. 10.1056/NEJMp2006141 32212516

[33] Stuckler D Basu S Suhrcke M Coutts A McKee M. The public health effect of economic crises and alternative policy responses in Europe: an empirical analysis. Lancet Lond Engl. (2009) 374:315–23. 10.1016/S0140-6736(09)61124-7 19589588

[34] Kawohl W Nordt C. COVID-19, unemployment, and suicide. Lancet Psychiatry. (2020) 7:389–90. 10.1016/S2215-0366(20)30141-3 32353269 PMC7185950

[35] Vardavas CI Nikitara K. COVID-19 and smoking: a systematic review of the evidence. Tob Induc Dis. (2020) 18:20. 10.18332/tid/119324 32206052 PMC7083240

[36] Saengow U Assanangkornchai S Casswell S. Alcohol: a probable risk factor of COVID-19 severity. Addiction. (2021) 116:204–5. 10.1111/add.15194 32688440 PMC7404678

[37] Gunnell D Appleby L Arensman E Hawton K John A Kapur N. Suicide risk and prevention during the COVID-19 pandemic. Lancet Psychiatry. (2020) 7:468–71. 10.1016/S2215-0366(20)30171-1 32330430 PMC7173821

