# Supplementary material for: Factors Associated With Mental Health Symptoms During the COVID-19 Pandemic in Hong Kong
Source: Front Psychiatry. 2021 Jun 7;12:617397. doi: 10.3389/fpsyt.2021.617397 (PMC8215194; doi:10.3389/fpsyt.2021.617397)
Supplement: Supplementary file 1 [file Table_1.docx]

**Supplementary Table. Mental health symptoms by shortage of preventive materials, perceptions of the outbreak, and change in income, smoking and alcohol consumption since the outbreak.**

|  | **Stress score Adjusted β (95%CI)** | | **Anxiety symptom Adjusted OR (95%CI)** | | **Depressive symptom Adjusted OR (95%CI)** | |
| --- | --- | --- | --- | --- | --- | --- |
|  | **Model 1 ^a^** | **Model 3 ^b^** | **Model 1 ^a^** | **Model 3 ^b^** | **Model 1 ^a^** | **Model 3 ^b^** |
| **Shortage of preventive materials** |  |  |  |  |  |  |
| Facemasks | 0.95 (0.69, 1.21) ^***^ | 0.82 (0.56, 1.10) ^***^ | 2.67 (1.93, 3.68) ^***^ | 2.53 (1.80, 3.56) ^***^ | 2.64 (1.90, 3.68) ^***^ | 2.26 (1.59, 3.22) ^***^ |
| Alcohol-based hand sanitizers | 1.30 (0.99, 1.61) ^***^ | 1.17 (0.85, 1.50) ^***^ | 2.30 (1.58, 3.34) ^***^ | 2.07 (1.37, 3.14) ^***^ | 2.41 (1.64, 3.53) ^***^ | 1.83 (1.20, 2.80) ^***^ |
| Cleaning products (e.g., bleach) | 0.83 (0.39, 1.27) ^***^ | 0.57 (0.13, 1.01) ^***^ | 3.81 (2.39, 6.06) ^***^ | 3.28 (2.03, 5.32) ^***^ | 3.39 (2.11, 5.46) ^***^ | 2.65 (1.62, 4.36) ^***^ |
| **No. of the types of shortage materials** |  |  |  |  |  |  |
| None | - | - | - | - | - |  |
| 1 | 0.60 (0.28, 0.92) ^***^ | 0.46 (0.15, 0.78) ^**^ | 2.07 (1.38, 2.11) ^***^ | 1.87 (1.23, 2.83) ^**^ | 1.82 (1.19, 2.79) ^**^ | 1.58 (1.02, 2.44) ^*^ |
| 2 | 1.39 (1.00, 1.78) ^***^ | 1.32 (0.91, 1.74) ^***^ | 1.59 (0.94, 2.71) | 1.50 (0.84, 2.66) | 2.00 (1.19, 3.35) ^**^ | 1.54 (0.87, 2.72) |
| 3 | 1.29 (0.74, 1.83) ^***^ | 1.13 (0.59, 1.68) ^**^ | 7.17 (4.07, 12.64) ^***^ | 6.53 (3.61, 11.80) ^***^ | 6.05 (3.40,10.77) ^***^ | 4.81 (2.63, 8.77) ^***^ |
| *P for trend* | <0.001 | <0.001 | <0.001 | <0.001 | <0.001 | <0.001 |
| **Perceptions of the outbreak** |  |  |  |  |  |  |
| I feel at risk of getting infected in the coming 6 months | 0.18 (0.08, 0.28) ^***^ | 0.15 (0.05, 0.25) ^**^ | 1.56 (1.37, 1.78) ^***^ | 1.55 (1.35, 1.77) ^***^ | 1.42 (1.24, 1.62) ^***^ | 1.38 (1.20, 1.58) ^***^ |
| I think people around me pose a threat to me | 0.32 (0.20, 0.44) ^***^ | 0.35 (0.23, 0.46) ^***^ | 1.47 (1.24, 1.74) ^***^ | 1.49 (1.25, 1.76) ^***^ | 1.63 (1.37, 1.95) ^***^ | 1.61 (1.34, 1.92) ^***^ |
| I feel the outbreak greatly affected daily life | 0.31 (0.19, 0.42) ^***^ | 0.37 (0.26, 0.49) ^***^ | 1.38 (1.17, 1.64) ^***^ | 1.45 (1.22, 1.73) ^***^ | 1.41 (1.19, 1.68) ^***^ | 1.45 (1.22, 1.73) ^***^ |
| I feel difficult to study/work at home | 0.27 (0.16, 0.38) ^***^ | 0.23 (0.13, 0.34) ^***^ | 1.60 (1.37, 1.87) ^***^ | 1.58 (1.35, 1.85) ^***^ | 1.41 (1.21, 1.64) ^***^ | 1.39 (1.19, 1.62) ^***^ |
| I feel inefficient studying/working at home | 0.33 (0.22, 0.45) ^***^ | 0.30 (0.19, 0.41) ^***^ | 1.62 (1.38, 1.91) ^***^ | 1.58 (1.34, 1.87) ^***^ | 1.71 (1.44, 2.02) ^***^ | 1.65 (1.40, 1.96) ^***^ |
| **Change in income since the outbreak ^c^** | |  |  |  |  |  |
| No change | - | - | - | - | - | - |
| Small reduction | 0.44 (0.11, 0.76) ^**^ | 0.28 (-0.05, 0.60) | 3.41 (2.00, 5.82) ^***^ | 3.14 (1.83, 5.39) ^***^ | 1.84 (0.23, 3.01) ^*^ | 1.68 (1.02, 2.78) ^*^ |
| Reduction by half | 0.55 (0.14, 0.97) ^**^ | 0.43 (0.02, 0.84) ^*^ | 4.30 (2.35, 7.90) ^***^ | 4.02 (2.18, 7.41) ^***^ | 2.62 (1.49, 4.63) ^***^ | 2.45 (1.38, 4.34) ^**^ |
| Large reduction | 0.81 (0.41, 1.21) ^***^ | 0.82 (0.43, 1.22) ^***^ | 4.52 (2.50, 8.16) ^***^ | 4.50 (2.48, 8.16) ^***^ | 2.47 (1.41, 4.32) ^***^ | 2.42 (1.37, 4.26) ^**^ |
| Unemployed | 1.69 (0.14, 2.24) ^***^ | 1.54 (0.99, 2.08) ^***^ | 5.94 (2.93, 12.04) ^***^ | 5.38 (2.64, 10.96) ^***^ | 3.94 (2.03, 7.66) ^***^ | 3.45 (1.75, 6.80) ^***^ |
| *P for trend* | <0.001 | <0.001 | <0.001 | <0.001 | <0.001 | <0.001 |

Note: Abbreviations: CI, confidence interval; OR, odds ratio.

^a^ Regression model 1: adjusted for sociodemographic variables including sex, age, education attainment,

^b^ Regression model 3: additionally adjusted for personal protection and social distancing measures

^c^ Excluded students, homemakers, and retirees, ^*^ P<0.05, ^**^ P<0.01, ^***^ P<0.001
